# Supplementary material for: Topological links in predicted protein complex structures reveal limitations of AlphaFold
Source: Commun Biol. 2023 Oct 28;6:1098. doi: 10.1038/s42003-023-05489-4 (PMC10613300; doi:10.1038/s42003-023-05489-4)
Supplement: Supplementary file 2 — Supplementary Information [file 42003_2023_5489_MOESM2_ESM.pdf]

Supplementary Information for the article "Topological Links in Predicted Protein Complex Structures Reveal Limitations of AlphaFold".

Yingnan Hou[1,2], Tengyu Xie[1,2], Liuqing He[1,3], Liang Tao[1,3], and Jing Huang[1,2 *]

[1] Key Laboratory of Structural Biology of Zhejiang Province, School of Life Sciences, Westlake University, 18 Shilongshan Road, Hangzhou 310024, Zhejiang, China.

[2] Westlake AI Therapeutics Lab, Westlake Laboratory of Life Sciences and Biomedicine, 18 Shilongshan Road, Hangzhou 310024, Zhejiang, China.

[3] Center for Infectious Disease Research, Westlake Laboratory of Life Sciences and Biomedicine, 18 Shilongshan Road, Hangzhou 310024, Zhejiang, China.

*Corresponding author: Jing Huang, huangjing@westlake.edu.cn

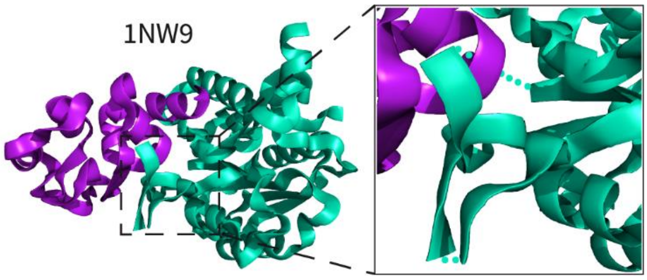

Supplementary Figure 1. A broken chain (in cyan) at the interaction interface causes a false topological link in the experimental structure (PDB code: 1NW9).

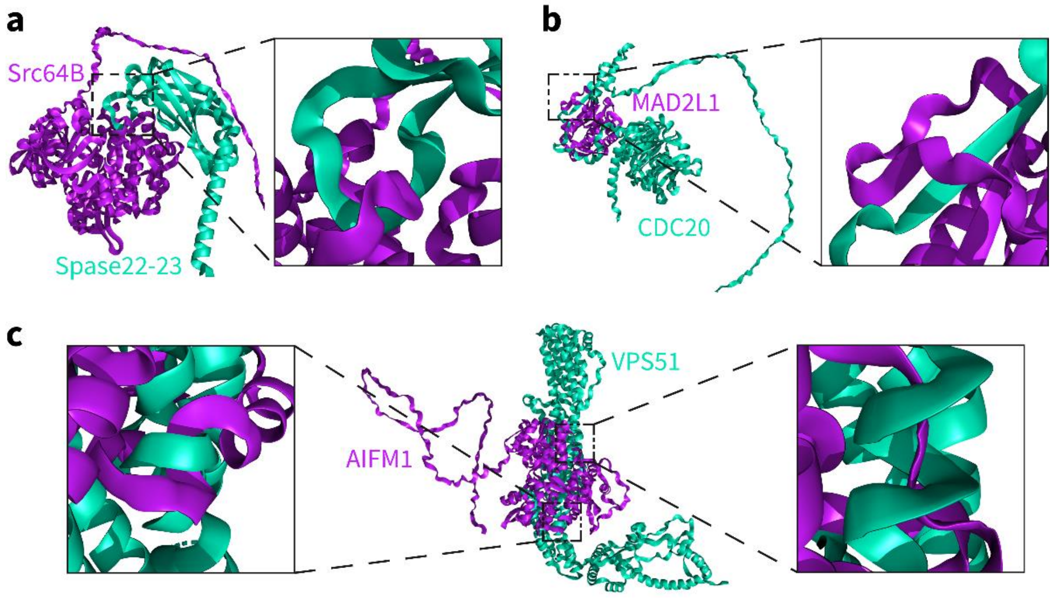

Supplementary Figure 2. Topologically linked structures in the two PPIs datasets predicted by AlphaFold-Multimer (v2.2.0). The gene names of the proteins are marked. a) Src64B-Spase22-23-1. b) MAD2L1-CDC20-1. c) AIFM1-VPS51-17: 10 topological links were identified in this structure.

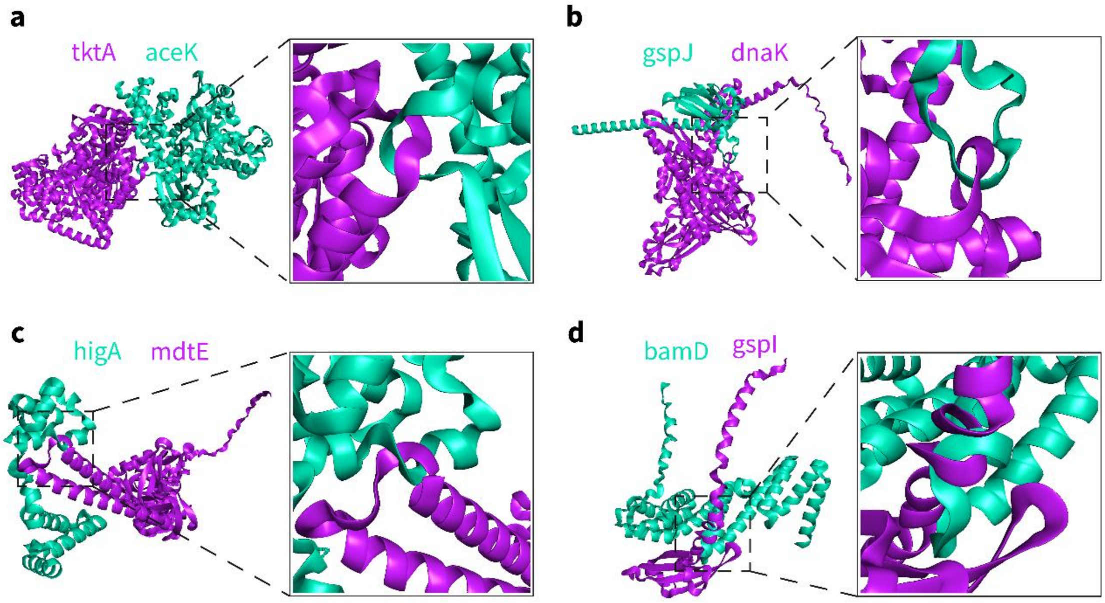

Supplementary Figure 3. Topologically linked structures of protein–protein complexes in *E. coli* predicted by AlphaFold-Multimer (v2.2.0). The gene names of the proteins are marked. a) tktA-aceK-1. b) dnaK-gspJ-1. c) mdtE-higA-2. d) gspI-bamD-2.

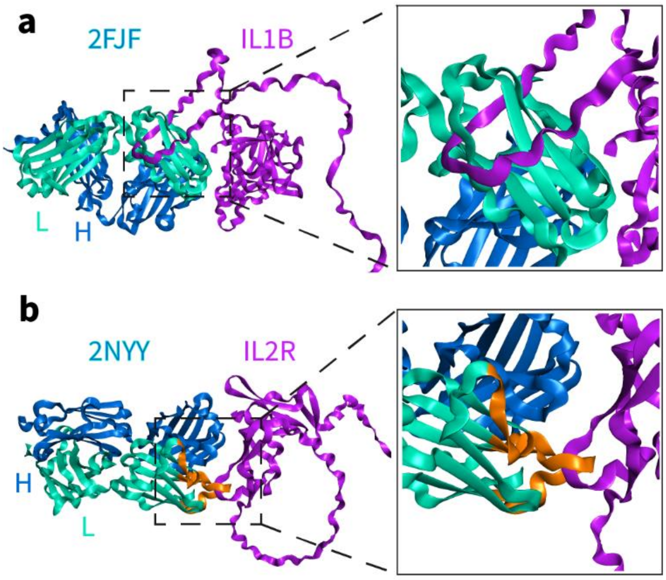

Supplementary Figure 4. Topologically linked structures of antibody–interleukin complexes in *Homo sapiens* predicted by AlphaFold-Multimer (v2.2.0) with the highest confidence scores. The PDB codes of the antibodies and the gene names of the interleukins are provided, with L indicating a light chain and H indicating a heavy chain. a) 2FJF-IL1B-1. b) 2nyy-IL2R-1. The CDR loops in the chain where topological links occur are colored orange.

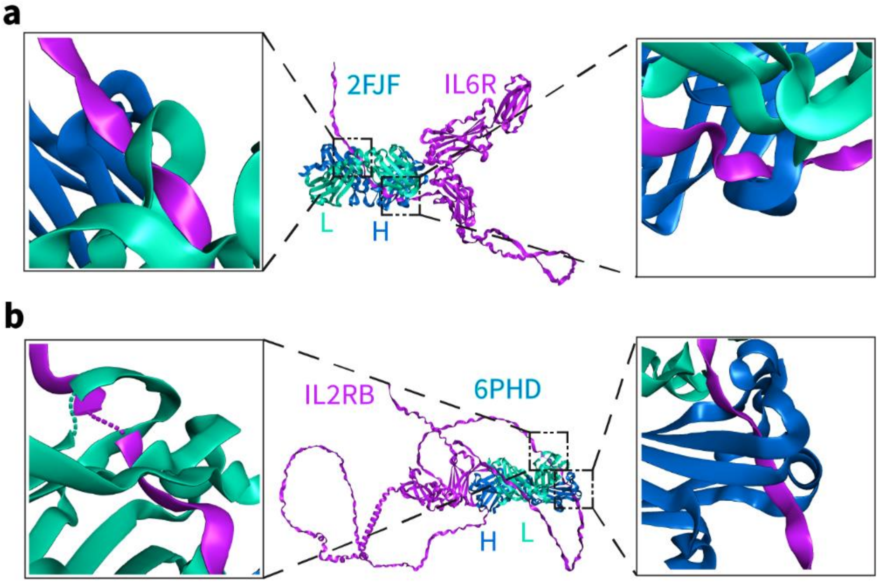

Supplementary Figure 5. Topologically linked structures of antibody–interleukin complexes in *Homo sapiens* predicted by AlphaFold-Multimer (v2.2.0) with more than one topological link. The interleukins form topological links with both the heavy chain (H) and the light chain (L) of the antibodies. The PDB codes of the antibodies and the gene names of the interleukins are provided. a) 2FJF-IL6R-12. b) 6PHD-IL2RB-6.

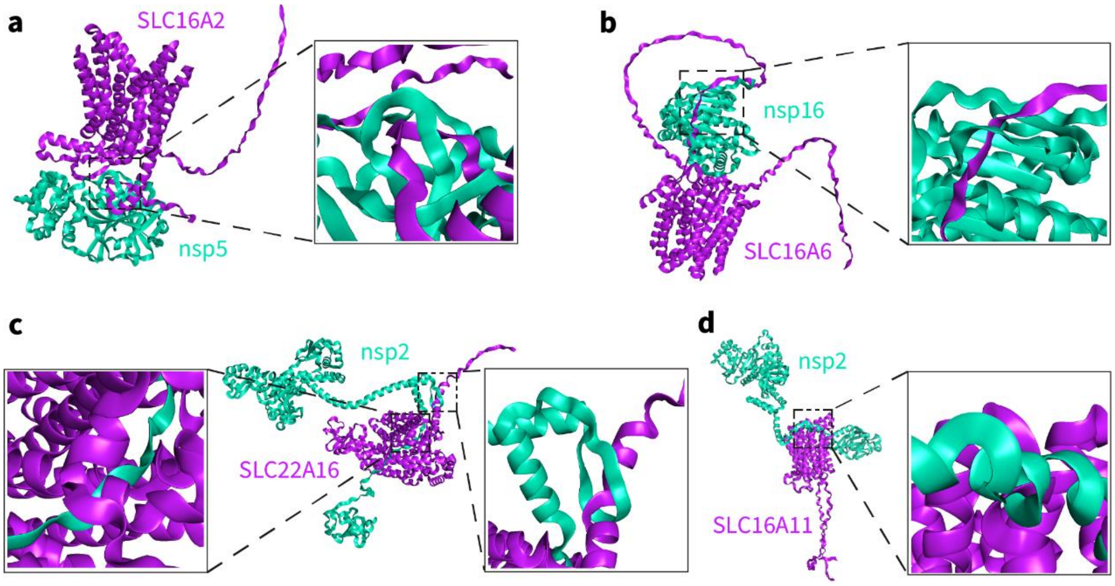

Supplementary Figure 6. Topologically linked structures of complexes formed by human MFS transporters and NSPs of SARS-CoV-2 predicted by AlphaFold-Multimer (v2.2.0). The gene names of the human MFS transporters (purple) and the NSPs (cyan) are provided. a) SLC16A2-nsp5-1. b) SLC16A6-nsp16-1. c) SLC22A16-nsp2-1. d) SLC16A11-nsp2-1.

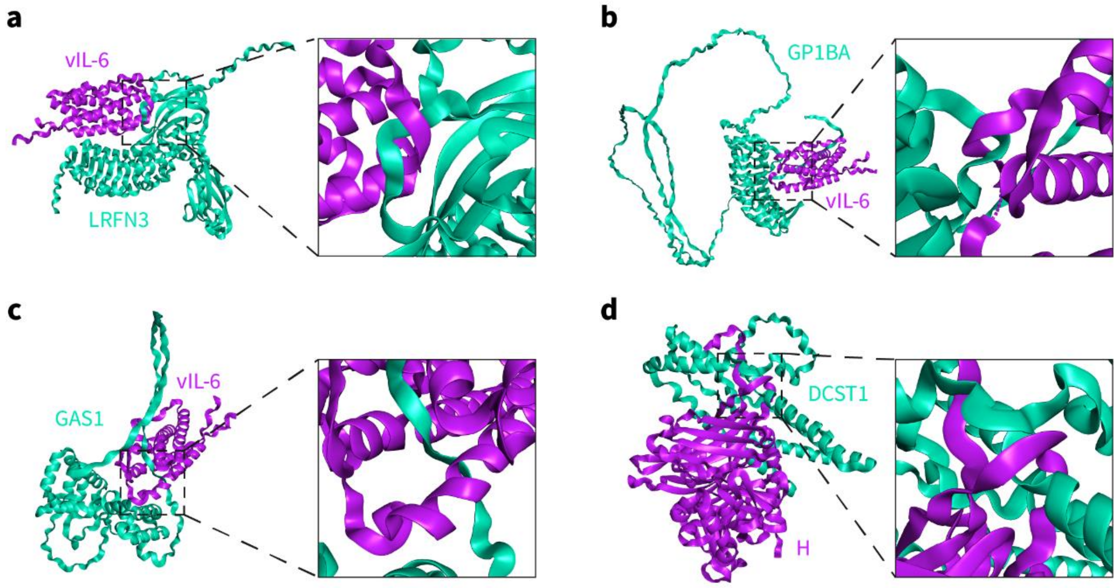

Supplementary Figure 7. Topologically linked structures of pathogenic virus and human membrane protein complexes predicted by AlphaFold-Multimer (v2.2.0). The gene names of human herpesvirus interleukin-6 homolog protein (vIL-6, purple), measles virus hemagglutinin glycoprotein (H, purple) and human membrane proteins (cyan) are provided. a) vIL-6-LRFN3-1. b) vIL-6-GP1BA-1. c) vIL-6-GAS1-2. d) H-DCST1-14.

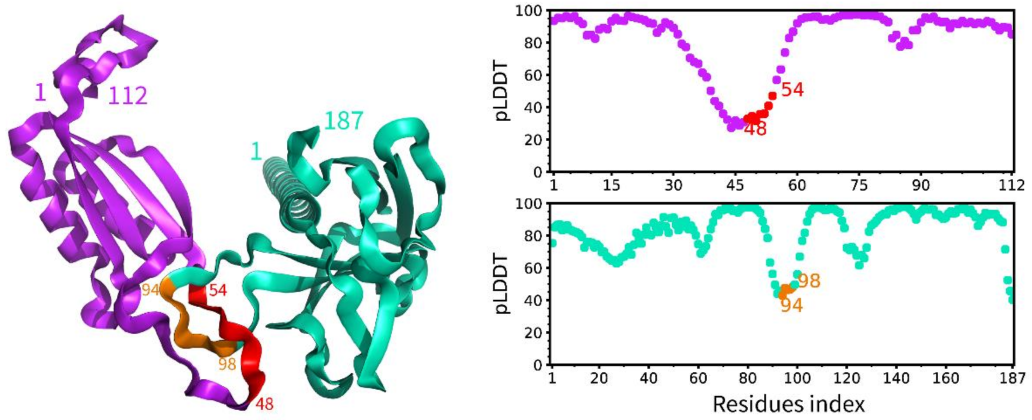

Supplementary Figure 8. The confidence scores (pLDDT) of atoms provided by AlphaFold-Multimer (v2.2.0) are relatively low where topological links occur in the exemplary structure (glnB-gspJ-4).

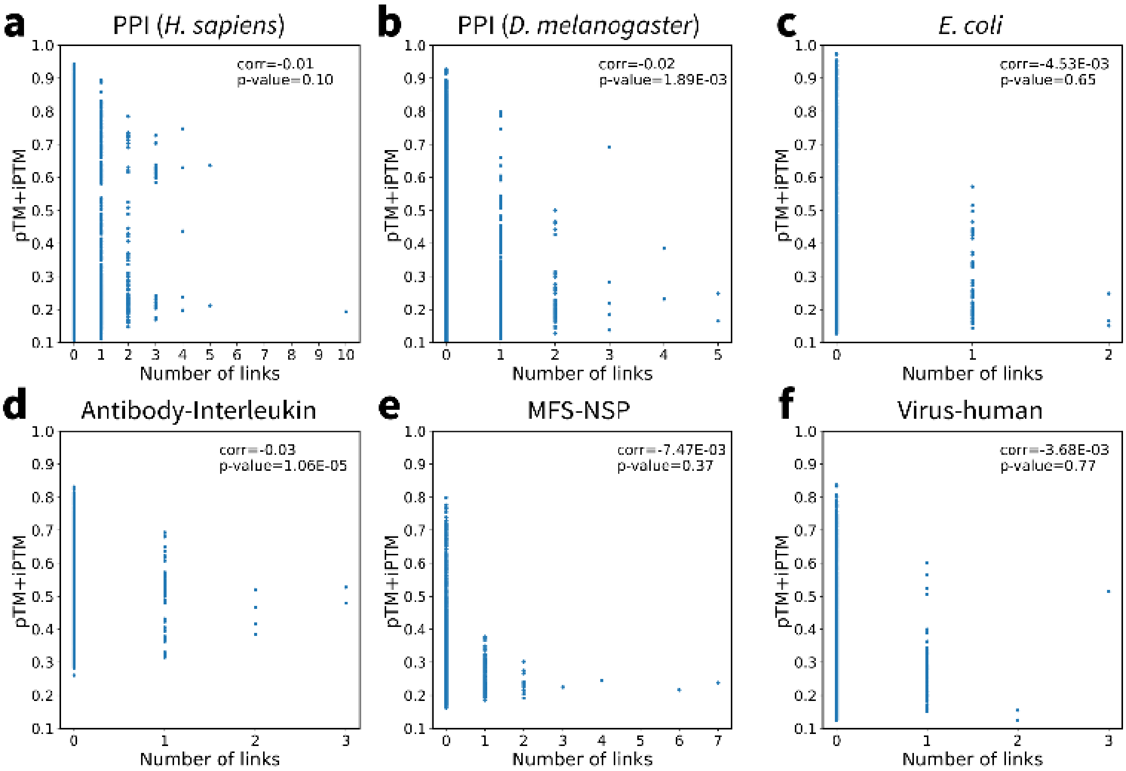

Supplementary Figure 9. The correlation between the number of topological links and the quality of predicted structures in term of the overall pTM+iPTM of predicted structures for the six datasets. The correlation coefficients and the p-values (with non-zero correlation as the alternative hypothesis) are provided, respectively.

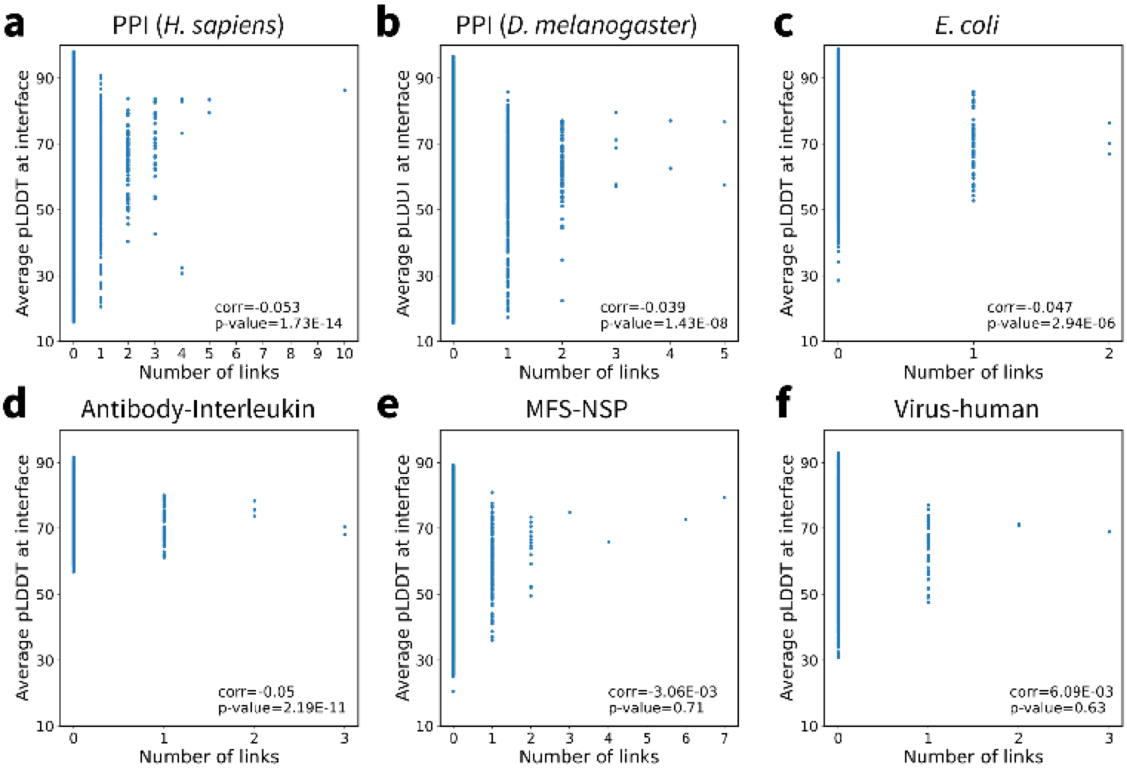

Supplementary Figure 10. The correlation between the number of topological links and the quality of predicted structures in term of the average pLDDT score at interface of predicted structures for the six datasets. The correlation coefficients and the p-values (with non-zero correlation as the alternative hypothesis) are provided, respectively.

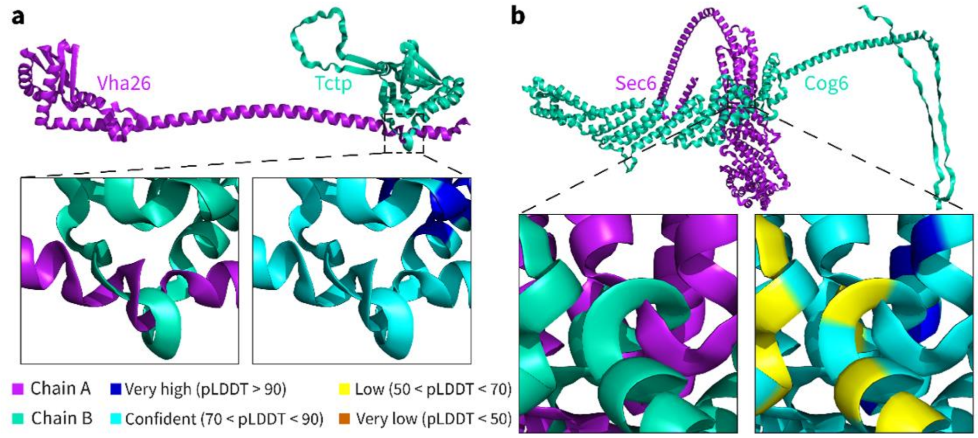

Supplementary Figure 11. Two exemplary topologically linked structures with confident average pLDDT score at interface of predicted structures. a) Vha26-Tctp-25 and b) Sec6-Cog6-1.

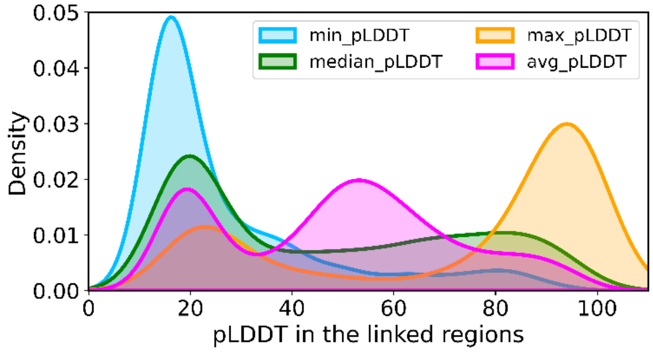

Supplementary Figure 12. Distributions of the maximal, minimal, median, and average pLDDT scores in the topologically linked regions, calculated from 1,558 topologically linked structures predicted by AlphaFold-Multimer. The topological linked region refers to the core region recognized by the algorithm, as well as the region encompassing six neighboring residues.

Supplementary Table 1. Comparison of methods for identifying topological links on the 4 AlphaFold-Multimer (AF2) [1] predicted structures and 4 experimental structures within labeled chains. Our method calculates and returns the numbers of topological links (NTLs) of these structures. The GLN method [2] was extended to apply to the 8 protein complexes, and the calculation was based on the GLN function in the topoly package [3]. The max|GLN| value represents the maximal absolute GLN value between one chain and all fragments of the other chain, and whGLN represents the overall GLN value of the whole chain. The calculations with LinkProt were executed via its web server; for details regarding Hopf links with different subtypes depending on chirality and chain orientation, refer to [4].

Structures	Source	Pair of chains	NTL	max|GLN|	whGLN	LinkProt	
						type	probability
glnB-gspJ-4	AF2	A-B	1	1	-0.943	Hopf.2	100%
panD-glnK-10	AF2	A-B	1	0.973	**0.117**	**unlink**	100%
fucO-bamD-1	AF2	A-B	1	1.149	-1.031	Hopf.2	100%
cysK-yoeB-1	AF2	A-B	1	1.087	0.93	Hopf.1	100%
1A73	PDB	A-B	0	0.817	0.723	Hopf.1	100%
1AV1	PDB	A-B	0	0.839	-0.713	Hopf.2	100%
2A68	PDB	A-B	0	0.934	0.934	Hopf.1	100%
5AUR	PDB	A-C	0	0.813	0.754	Hopf.1	100%

Supplementary Table 2. Summary of the number of topologically linked structures, characterized by distinct values of the average pLDDT scores at the interface and the overall pTM+iPTMs.

Dataset	The average pLDDT score at interface				pTM+iPTM		
	High ($x>90$)	Confident ($70<x<90$)	Low ($50<x<70$)	Very low ($x<50$)	Confident ($x>0.7$)	Low ($0.5<x<0.7$)	Very low ($x<0.5$)
PPI of *H. sapiens*	2	214	334	91	68	66	507
PPI of *D. melanogaster*	0	126	335	104	4	20	541
E. coli	0	31	29	0	0	2	58
Antibody-interleukin	0	37	28	0	0	38	27
MFS-NSP	0	30	115	24	0	0	169
Virus-human	0	16	39	3	0	5	53
Total	2	454	880	222	72	131	1355

Supplementary Table 3. The number and the percentage of predicted structures with pDockQ > 0.5, as well as those containing topological links. The percentage of topological linked structures for those with pDockQ > 0.5 is also reported.

Dataset	All predicted structures with pDockQ > 0.5		Topologically linked structures with pDockQ > 0.5		Percentage of structures with pDockQ > 0.5 containing links
	No.	Percentage	No.	Percentage	
PPI of *H. sapiens*	5483	26.08%	271	42.28%	4.94%
PPI of *D. melanogaster*	2488	12.02%	156	27.61%	6.27%
E. coli	555	5.55%	19	31.67%	3.42%
Antibody-interleukin	25	0.14%	3	4.62%	12.00%
MFS-NSP	148	1.03%	33	19.53%	22.30%
Virus-human	137	2.13%	10	17.24%	7.30%
Total	8836	9.76%	492	31.58%	5.57%

Supplementary Table 4. Statistical analysis on the performance of our method and LinkProt on a benchmark dataset of 306 protein complex structures, including 200 AlphaFold-Multimer predicted structures and 106 experimental structures. Specifically, the 200 AlphaFold-Multimer predicted structures were identified as containing topological links by our method and filtered with an average pLDDT score at their interaction interfaces no less than 70. Similarly, the 106 experimental structures were gathered from the LinkProt database and filtered with the criterion of two chains being classified as probabilistic links (excluding deterministic and macromolecular links, as we focused on non-covalent bond-formed topological links). The presence or absence of topological links in each structure was confirmed through manual inspection to determine the ground truth, resulting in 203 structures containing topological links and 103 without. The numbers of true positives (TP), false positives (FP), false negatives (FN), and true negatives (TN) are reported, together with sensitivity, specificity, precision and False Discovery Rate (FDR). TP represents the

number of correctly identified topologically linked structures, while FP indicates structures that were falsely identified as linked. FN refers to structures that were mistakenly identified as unlinked, and TN denotes the appropriately identified unlinked structures.

Method	TP	FP	FN	TN	Sensitivity	Specificity	Precision	FDR
this work	203	0	3	100	100.00%	97.09%	98.54%	1.46%
LinkProt	183	20	103	0	90.15%	0.00%	63.99%	36.01%

Supplementary Table 5. Summary of link detection by our method on the datasets of AlphaFold-Multimer (v2.1.0) predicted structures. Note that AlphaFold-Multimer generates 5 predictions for each protein pair.

Species		All predicted structures			Top-ranked structures		
Bait protein	Prey protein	Total	Linked	Percentage	Total	Linked	Percentage
E. coli	*E. coli*	2000	227	11.35%	400	26	6.50%
H. sapiens	SARS-CoV-2	2870	1449	50.49%	574	222	38.68%
Human herpesvirus	*H. sapiens*	1060	135	12.74%	212	15	7.08%
total		5930	1811	30.54%	1186	263	22.18%

Supplementary References

1 Evans, R. *et al.* Protein complex prediction with AlphaFold-Multimer. *BioRxiv* (2021).

2 Niemyska, W., Millett, K. C. & Sulkowska, J. I. GLN: a method to reveal unique properties of lasso type topology in proteins. *Scientific reports* **10**, 1-12 (2020).

3 Dabrowski-Tumanski, P., Rubach, P., Niemyska, W., Gren, B. A. & Sulkowska, J. I. Topoly: Python package to analyze topology of polymers. *Briefings in Bioinformatics* **22**, bbaa196 (2021).

4 Dabrowski-Tumanski, P. *et al.* LinkProt: a database collecting information about biological links. *Nucleic acids research*, gkw976 (2016).

5 Vreven, T. *et al.* Updates to the integrated protein–protein interaction benchmarks: docking benchmark version 5 and affinity benchmark version 2. *Journal of molecular biology* **427**, 3031-3041 (2015).
