# Supplementary material for: Topological links in predicted protein complex structures reveal limitations of AlphaFold
Source: Commun Biol. 2023 Oct 28;6:1098. doi: 10.1038/s42003-023-05489-4 (PMC10613300; doi:10.1038/s42003-023-05489-4)
Supplement: Supplementary file 3 — Description of additional supplementary files [file 42003_2023_5489_MOESM3_ESM.docx]

Description of Additional Supplementary Files

**File name:** Supplementary Data 1

**Description:** The detection of topological links in the experimental structures from the protein‒protein docking benchmark DB5.0 set (DB5.0).

**File name:** Supplementary Data 2

**Description:** The detection of topological links in the 22,003 high-quality experimental structures from the PDB (expPDBs).

**File name:** Supplementary Data 3

**Description:** Information on the proteins in the datasets generated in this work.

**File name:** Supplementary Data 4

**Description:** Detailed information about the benchmark dataset of 306 protein complex structures.

**File name:** Supplementary Data 5

**Description:** The source data behind the Figure 3 in the paper.
