# Supplementary material for: Topological links in predicted protein complex structures reveal limitations of AlphaFold
Source: Commun Biol. 2023 Oct 28;6:1098. doi: 10.1038/s42003-023-05489-4 (PMC10613300; doi:10.1038/s42003-023-05489-4)
Supplement: Supplementary file 9 — Reporting Summary [file 42003_2023_5489_MOESM9_ESM.pdf]

nature portfolio

Corresponding author(s): Jing Huang

Last updated by author(s): Oct 8, 2023

Reporting Summary

Nature Portfolio wishes to improve the reproducibility of the work that we publish. This form provides structure for consistency and transparency in reporting. For further information on Nature Portfolio policies, see our Editorial Policies and the Editorial Policy Checklist.

Statistics

For all statistical analyses, confirm that the following items are present in the figure legend, table legend, main text, or Methods section.

n/a	Confirmed	
☐	☒	The exact sample size (*n*) for each experimental group/condition, given as a discrete number and unit of measurement
☐	☒	A statement on whether measurements were taken from distinct samples or whether the same sample was measured repeatedly
☐	☒	The statistical test(s) used AND whether they are one- or two-sided<br>*Only common tests should be described solely by name; describe more complex techniques in the Methods section.*
☒	☐	A description of all covariates tested
☒	☐	A description of any assumptions or corrections, such as tests of normality and adjustment for multiple comparisons
☐	☒	A full description of the statistical parameters including central tendency (e.g. means) or other basic estimates (e.g. regression coefficient) AND variation (e.g. standard deviation) or associated estimates of uncertainty (e.g. confidence intervals)
☐	☒	For null hypothesis testing, the test statistic (e.g. *F*, *t*, *r*) with confidence intervals, effect sizes, degrees of freedom and *P* value noted<br>*Give P values as exact values whenever suitable.*
☒	☐	For Bayesian analysis, information on the choice of priors and Markov chain Monte Carlo settings
☒	☐	For hierarchical and complex designs, identification of the appropriate level for tests and full reporting of outcomes
☒	☐	Estimates of effect sizes (e.g. Cohen's *d*, Pearson's *r*), indicating how they were calculated

Our web collection on statistics for biologists contains articles on many of the points above.

Software and code

Policy information about availability of computer code

Data collection	The protein sequences in this work were downloaded from the UniProt database (https://www.uniprot.org/), except that the sequences of the 40 antibodies were taken from the PDB (https://www.rcsb.org/), SARS-CoV-2 NSPs were taken from the NCBI database (https://www.ncbi.nlm.nih.gov/), human virus were taken from PDBe (https://www.ebi.ac.uk/pdbe/) and human membrane proteins were taken from the human protein atlas (https://www.proteinatlas.org/). AlphaFold-Multimer (https://github.com/deepmind/alphafold) in v2.2.0 and v2.1.0 are used to generate the predicted structures, with the corresponding databases: UniRef90 v2019_10 (https://ftp.ebi.ac.uk/pub/databases/uniprot/previous_releases/release308 2019_10/uniref/), BFD (https://bfd.mmseqs.com/), Uniclust30 v2018_08 (https://wwwuser.gwdg.de/~compbiol/uniclust/2018_08/), MGnify clusters v.2018_12 (https://ftp.ebi.ac.uk/pub/databases/metagenomics/peptide_database/2018_12/), UniProt (https://ftp.ebi.ac.uk/pub/databases/uniprot/current_release/knowledgebase/complete), "pdb_mmcif" (rsync.rcsb.org::ftp_data/structures/divided/mmCIF/ as of Feb 10, 2022). Experimental structures were downloaded from the PDB (https://www.rcsb.org/).
Data analysis	https://github.com/JingHuangLab/topoLink<br>PyMOL (https://pymol.org/2/)

For manuscripts utilizing custom algorithms or software that are central to the research but not yet described in published literature, software must be made available to editors and reviewers. We strongly encourage code deposition in a community repository (e.g. GitHub). See the Nature Portfolio guidelines for submitting code & software for further information.

Data

Policy information about availability of data

All manuscripts must include a data availability statement. This statement should provide the following information, where applicable:

- Accession codes, unique identifiers, or web links for publicly available datasets
- A description of any restrictions on data availability
- For clinical datasets or third party data, please ensure that the statement adheres to our policy

The sequence of each protein from the six datasets and the source code for detecting topologically linked structures for protein-protein complexes are available at https://github.com/JingHuangLab/topoLink. Supplementary Data 1 is a .xlsx file that includes data for reproducing the GLN matrices in Figure 3. Additional information is available from the corresponding author upon request.

Human research participants

Policy information about studies involving human research participants and Sex and Gender in Research.

Reporting on sex and gender	Not applicable.
Population characteristics	Not applicable.
Recruitment	Not applicable.
Ethics oversight	Not applicable.

Note that full information on the approval of the study protocol must also be provided in the manuscript.

Field-specific reporting

Please select the one below that is the best fit for your research. If you are not sure, read the appropriate sections before making your selection.

☒ Life sciences ☐ Behavioural & social sciences ☐ Ecological, evolutionary & environmental sciences

For a reference copy of the document with all sections, see nature.com/documents/nr-reporting-summary-flat.pdf

Life sciences study design

All studies must disclose on these points even when the disclosure is negative.

Sample size	Six datasets were generated using AlphaFold-Multimer v2.2.0, including 21,025 predicted structures for 841 protein pairs from Homo sapiens, 20,700 predicted structures for 828 protein pairs from Drosophila melanogaster, 10,000 predicted structures for the combination of 20 PPIs from E. coli, 18,000 predicted structures for the combination of 40 human antibodies and 18 human interleukins, 14,350 predicted structures for the combinations of 41 major facilitator superfamily (MFS) proteins and 14 nonstructural proteins (NSPs) of SARS-CoV-2, and 6,425 predicted structures for 257 human virus and human membrane protein complexes, respectively. Our method also applied to a filtered set of 22,003 high-quality experimental complex structures from the PDB.
Data exclusions	The two PPIs datasets were obtained from the positive PPIs benchmark datasets and originally collected from the the public Database of Interacting Proteins, which were filtered to include protein pairs with less than 1536 total residues as suggested in AlphaFold-Multimer, and excluded one protein pair whose structural prediction failed.
Replication	Not applicable.
Randomization	Not applicable.
Blinding	Not applicable.

Reporting for specific materials, systems and methods

We require information from authors about some types of materials, experimental systems and methods used in many studies. Here, indicate whether each material, system or method listed is relevant to your study. If you are not sure if a list item applies to your research, read the appropriate section before selecting a response.

Materials & experimental systems

n/a	Involved in the study
☒	☐ Antibodies
☒	☐ Eukaryotic cell lines
☒	☐ Palaeontology and archaeology
☒	☐ Animals and other organisms
☒	☐ Clinical data
☒	☐ Dual use research of concern

Methods

n/a	Involved in the study
☒	☐ ChIP-seq
☒	☐ Flow cytometry
☒	☐ MRI-based neuroimaging
